# *In Vitro* Immunomodulatory Activity of a Transition-State Analog Inhibitor of Human Purine Nucleoside Phosphorylase in Cutaneous Leishmaniasis

**DOI:** 10.1155/2017/3062892

**Published:** 2017-08-27

**Authors:** Natália Barbosa Carvalho, Fernanda Ventin de Oliveira Prates, Rafael de Castro da Silva, Mayra Elizabeth Ferreira Dourado, Camila Farias Amorim, Paulo Roberto Lima Machado, Fernanda Grendene Pacheco, Temis Weber Furlanetto Corte, Pablo Machado, Diógenes Santiago Santos, Edgar Marcelino de Carvalho

**Affiliations:** ^1^Serviço de Imunologia do Complexo Hospitalar Universitário Professor Edgard Santos (Com-HUPES), Universidade Federal da Bahia, Salvador, BA, Brazil; ^2^Centro de Pesquisas em Biologia Molecular e Funcional (CPBMF), Pontifícia Universidade Católica do Rio Grande do Sul, Porto Alegre, RS, Brazil; ^3^Fundação Oswaldo Cruz (FIOCRUZ), Instituto Gonçalo Moniz (CPqGM), Salvador, BA, Brazil

## Abstract

Cutaneous leishmaniasis (CL) is the most common clinical form of American tegumentary leishmaniasis caused by *Leishmania* (*Viannia*) *braziliensis*. CL is associated with a strong Th1 immune response. This exacerbated inflammatory response is correlated with severity of disease and delays the healing time of the ulcer. The fourth-generation immucillin derivative (DI4G), a potent inhibitor of purine nucleoside phosphorylase, has been proposed as a promising agent in the treatment of diseases associated with T cell activation. Herein, we evaluated the *in vitro* immunomodulatory activity of DI4G in cells of patients presenting with CL. Peripheral blood mononuclear cells (PBMC) from CL patients were stimulated with soluble leishmania antigen (SLA), in the presence or absence of DI4G, and IFN-*γ*, TNF, CXCL9, and CXCL10 levels were determined by ELISA. Lymphocyte proliferation in the presence or absence of DI4G was also evaluated, using flow cytometry. DI4G was able to decrease (*p* < 0.05) IFN-*γ* production but did not change the TNF, CXCL9, and CXCL10 levels. DI4G decreased (*p* < 0.05) the lymphoproliferative response mediated by CD8^+^ T cells, but not that by CD4^+^ T cells. DI4G is able to attenuate the exaggerated immune response in CL, exhibiting immunomodulatory activity in IFN-*γ* production and in CD8^+^ T cell proliferation.

## 1. Introduction

Leishmaniasis is a global health problem and it is considered one of the most important neglected tropical diseases. Cutaneous leishmaniasis (CL) is the most common clinical form of American tegumentary leishmaniasis (ATL). It is estimated that 0.7 million to 1.2 million new cases occur worldwide annually [1]. In Latin America, CL is caused mainly by the protozoan *Leishmania (Viannia) braziliensis* which is transmitted by the bite of infected female phlebotomine sandflies of the genus *Lutzomyia* [2]. The strong inflammatory response with infiltration of lymphocytes, macrophages, granuloma formation, and small numbers or absence of parasites within skin lesions is a hallmark of CL caused by *L. braziliensis* [3]. The cell-mediated immune response is considered the main defense mechanism against protozoan parasites. However, in CL, both CD4^+^ and CD8^+^ T cells have been associated with pathology [4–7]. The inflammatory cytokines, such as IFN-*γ* and TNF, are important for control of parasite replication, but an exaggerated Th1 immune response observed during *L. braziliensis* infection leads to the development of tissue injury [5, 8–11]. Regarding CD8^+^ T cells, it was documented that T cell-mediated cytotoxicity is higher in severe forms of the disease [12, 13]. Moreover, rather than leishmania killing, cytotoxicity mediated by CD8^+^ T cells is associated with an intense inflammatory response and killing of parasite-infected cells, rather than leishmania death [7, 13].

In Latin America, pentavalent antimony is the first-line drug for treatment of CL. However, a high rate of therapeutic failure has been documented [14–16]. More recently, it has been shown that the association of antimony with immunoregulatory drugs, such as the granulocyte and monocyte colony-stimulating factor (GM-CSF) or pentoxyfilline, a drug that reduces TNF levels, is more effective than antimony alone and reduces the healing time of cutaneous and mucosal leishmaniasis [16–20].

**Figure 1:**
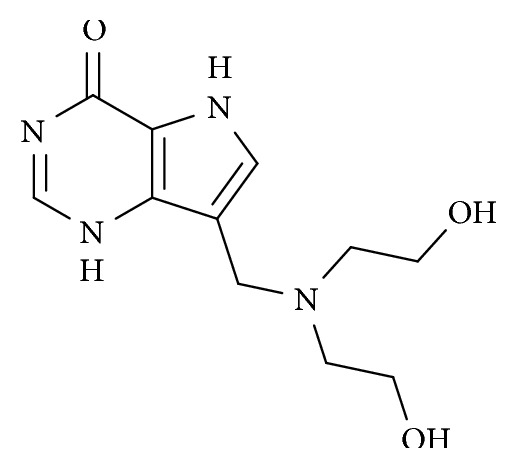
Chemical structure of the synthesized fourth-generation immucillin derivative (DI4G).

It is well documented that T cell proliferation and activation is dependent on the purine nucleotide phosphorylase (PNP) enzyme activity. PNP operates in catalyzing reversible phosphorylation of purine nucleoside leading to production of their respective bases and a phosphorylated pentose, an important reaction in the recycling of purines, allowing the synthesis of essential compounds for the high proliferative capacity observed in activated T cells [21]. The fourth-generation immucillin derivative DI4G (Figure 1) is a potent transition-state analog inhibitor of human PNP (HsPNP). This compound has been described as competitive inhibitor of HsPNP with *K*_i_ of 11.8 ± 1.47 nM regarding inosine substrate [22]. In addition, DI4G showed an IC_50_ value of 40.6 ± 0.36 nM when evaluated at substrate concentrations near to the *K*_M_ values for HsPNP [22]. Therefore, as pathology in CL is due to an exaggerated inflammatory response to leishmania antigen, we evaluated in the present study the *in vitro* immunomodulatory activity of DI4G in CL.

## 2. Material and Methods

### 2.1. Patients

This study included 15 CL patients followed at the Corte de Pedra's Health Post, in southeast Bahia, Brazil, an area for *L. braziliensis* transmission. Diagnosis of CL was based on the presence of a typical skin ulcer without mucosal involvement, associated with detection of *L. braziliensis* DNA by polymerase chain reaction in biopsied tissue, parasite isolation in culture, or identification of amastigotes in histopathology [23]. Immunologic studies were performed before therapy. The study was approved by the Ethics Committee of the Medical School of Federal University of Bahia and an informed consent was obtained from all participants.

### 2.2. Fourth-Generation Immucillin Derivative (DI4G)

The immucillin derivative DI4G was synthesized according to already described protocol [24]. The compounds had spectroscopic and spectrometric data that were in agreement with the proposed structure.

### 2.3. Peripheral Blood Mononuclear Cell (PBMC) Culture

Peripheral blood mononuclear cells (PBMCs) from CL patients were obtained from heparinized venous blood by density gradient centrifugation using Ficoll-Hypaque (GE Healthcare Bio-Sciences, Uppsala, Sweden). The cells were cultivated at 3 × 10^6^ cells/ml/well into 24-well plates in RPMI 1640 medium (Gibco BRL, Grand Island, NY), supplemented with 2 mM L-glutamine, 25 mM HEPES, 10% heat-inactivated fetal bovine serum (Sigma, St. Louis, MO), and 0.05% gentamicin at 10 mg/ml (Gibco BRL, Grand Island, NY). PBMCs were cultivated without stimulus, stimulated with soluble leishmania antigen (SLA; 5 *μ*g/ml), in the presence or absence of DI4G (300 nM), and phytohemagglutinin (PHA, 10 *μ*l/ml; Gibco BRL, Grand Island, NY) at 37°C in an atmosphere of 5% CO_2_. Culture supernatants were collected 72 hours after stimulation and stored at −20°C until used for cytokine measurement.

### 2.4. Cytokine Measurement

IFN-*γ*, TNF, CXCL9, and CXCL10 levels were determined in PBMC culture supernatants by enzyme-linked immunosorbent assay (ELISA) using a commercial kit and according to the manufacturer's instructions (BD Biosciences Pharmingen, San Jose, CA, USA).

### 2.5. Intracellular Cytokine Staining

For intracellular detection of IFN-*γ*, PBMCs were adjusted to 4 × 10^5^ cells/well into 96-well plates. The cells were stimulated with SLA (5 *μ*g/ml), SLA plus DI4G (300 nM), or anti-CD3/anti-CD28 (1 *μ*g/ml and 0.5 *μ*g/ml; BD Pharmingen, San Diego, CA) as positive control at 37°C for 20 h. Brefeldin A (10 *μ*g/ml; Sigma, St. Louis, MO) was added for the last 4 h of culture. Cells were then stained for surface markers and intracellular cytokine. Briefly, cells were stained with monoclonal antibodies (anti-CD3-FITC, anti-CD4-APC (eBioscience, San Diego, CA), anti-CD56-FITC, anti-CD3-PercP-Cy5.5, and anti-CD8-APC (BD Pharmingen, San Diego, CA)) for 20 minutes at 4°C. Cells were washed, fixed using a 2% formaldehyde solution, and permeabilized with a 0.5% saponin solution in phosphate-buffered saline (PBS). Cells were stained with anti-IFN-*γ*-PE (eBioscience, San Diego, CA) for 30 minutes at room temperature. Cells were washed with permeabilization solution and resuspended in PBS. Cells (150,000 events) were collected using a II FACSCanto flow cytometer (Becton Dickinson, San Jose, CA), and data were analyzed using the FlowJo Software version 7.6 (Tree Star, Ashland, OR). The lymphocyte and NK populations were selected according to size and granularity and analyzed in CD3^+^ and CD3^−^ gates, respectively.

### 2.6. Proliferation Assay

For proliferation assay, PBMCs were cultivated as described above. Six days after culture, cells were stained for surface markers (anti-CD3-FITC (eBioscience, San Diego, CA), anti-CD4-PE, and anti-CD8-APC (BD Pharmingen, San Diego, CA)) and intracellularly with anti-Ki-67-PercP-Cy5.5 (BD Pharmingen, San Diego, CA), a cellular marker for proliferation [25]. Cells were then fixed and acquisition and data analysis were performed as described above.

### 2.7. Statistical Analyses

GraphPad Prism 5 Software (San Diego, CA) was used to carry out the statistical evaluation. *p* value < 0.05 was considered to indicate a significant difference. Statistical analyses were performed using Wilcoxon *t*-test.

## 3. Results

### 3.1. Dose Response Curve to Determine the Optimal Concentration of DI4G

**Figure 2:**
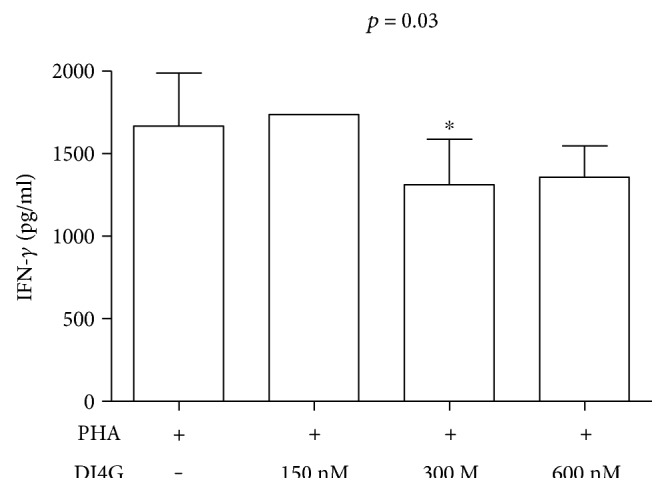
Dose response evaluation of DI4G. PBMCs from healthy individuals (*n* = 6) were stimulated with PHA (30 *μ*l/ml), in the absence and presence of HsPNP inhibitor (150 nM, 300 nM, and 600 nM), and levels of IFN-*γ* were determined by ELISA 72 h poststimulation. Results are presented as means ± standard deviations. Statistical analyses were performed using Wilcoxon *t*-test (^∗^*p* < 0.05).

To determine the concentration of DI4G that was capable of impairing cytokine production by activated T cells, PBMCs from healthy individuals were cultivated with the mitogen PHA (30 *μ*l/ml) in the absence or in the presence of different concentrations of DI4G (150 nM, 300 nM, and 600 nM). The optimal concentration chosen of compound was 300 nM and it rendered 21.3% reduction in IFN-*γ* production, *p* < 0.05 (Figure 2).

### 3.2. DI4G Impairs IFN-*γ* Production by PBMCs from CL Patients

**Figure 3:**
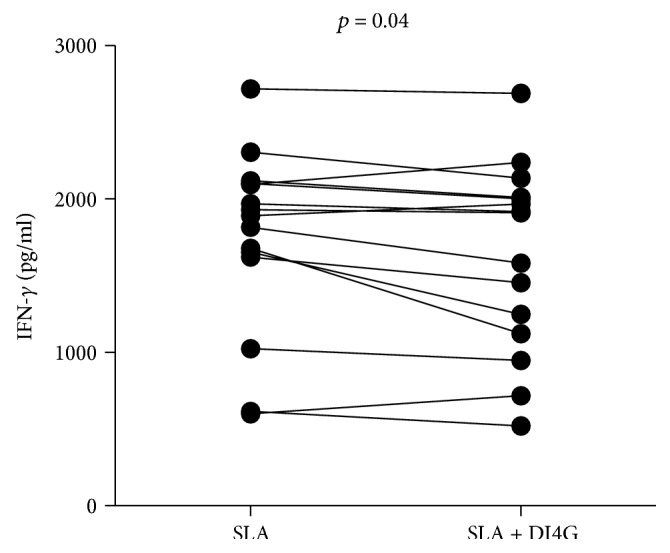
Modulatory activity of DI4G in IFN-*γ* production by PBMCs from CL patients. PBMCs from cutaneous leishmaniasis patients (*n* = 15) were cultured with SLA (5 *μ*g/ml), in the absence and presence of compound (300 nM), for 72 h. Levels of IFN-*γ* were determined by ELISA. Statistical analyses were performed using Wilcoxon *t*-test.

To evaluate the modulatory action of compound in cellular immune response, PBMCs from CL patients were stimulated with SLA (5 *μ*g/ml), in the presence and absence of HsPNP inhibitor (300 nM), and the levels of IFN-*γ*, TNF, CXCL9, and CXCL10 were measured by ELISA. IFN-*γ* production by stimulated cells cultured with DI4G was lower than that by cells stimulated with only SLA, *p* < 0.05 (Figure 3). Overall, there was a 6.4% reduction in IFN-*γ* production (weighted average, range 0–33%), indicating that compound may downmodulate the immune response in cutaneous leishmaniasis. When the production of TNF, CXCL9, and CXCL10 was evaluated, no differences were observed between stimulated cells in the absence and presence of HsPNP inhibitor (data not shown).

**Figure 4:**
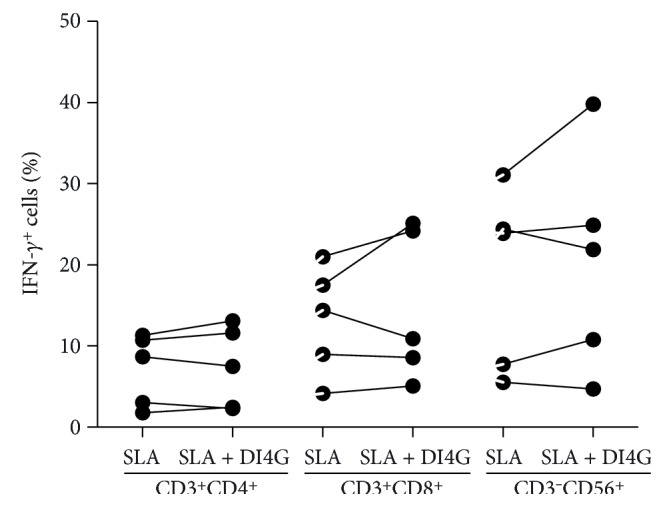
Frequencies of IFN-*γ*-producing cells from CL patients after SLA and DI4G stimulation. PBMCs from CL patients (*n* = 5) were stimulated with SLA (5 *μ*g/ml), in the absence and presence of compound (300 nM), for 20 h. Cells were stained for CD3, CD4, CD8, CD56, and IFN-*γ* and collected using II FACSCanto flow cytometer. To analyze the data, the lymphocyte and NK populations were selected according to size and granularity and analyzed in CD3^+^ gate and CD3^−^ gate, respectively. Statistical analyses were performed using Wilcoxon *t*-test.

In order to determine the influence of DI4G in IFN-*γ* producing cells, the frequencies of lymphocytes and NK cells expressing this cytokine were determined by flow cytometry. There were no differences in the numbers of CD4^+^ lymphocytes, CD8^+^ lymphocytes, and NK cells producing IFN-*γ* in the absence and presence of a molecule (Figure 4).

### 3.3. DI4G Is Able to Decrease CD8^+^ Lymphocyte Proliferation *In Vitro* from CL Patients

**Figure 5:**
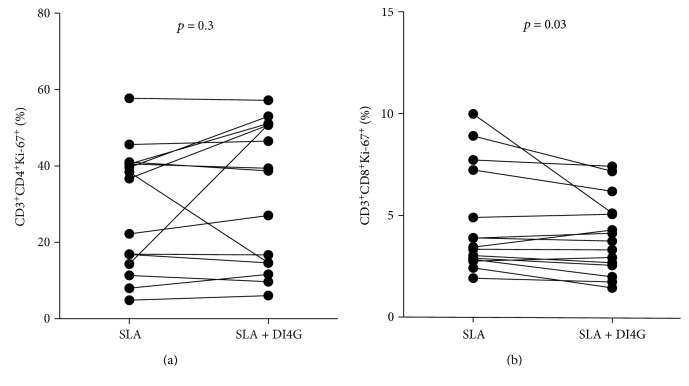
Modulatory activity of DI4G in proliferation of PBMCs from CL patients. PBMCs from cutaneous leishmaniasis patients (*n* = 15) were cultured with SLA (5 *μ*g/ml), in the absence and presence of HsPNP inhibitor (300 nM), for 6 days. CD4^+^ T cells (a) and CD8^+^ T cells (b) were stained for CD3, CD4, CD8, and Ki-67 and collected using II FACSCanto flow cytometer. To analyze the data, the lymphocyte populations were selected according to size and granularity and analyzed in CD3^+^ gate. Statistical analyses were performed using Wilcoxon *t*-test.

In order to evaluate the action of DI4G in the proliferation of lymphocytes, PBMCs from CL patients were cultured for 6 days with SLA (5 *μ*g/ml), in the presence and absence of compound (300 nM). There was an impairment in the lymphoproliferative response mediated by CD8^+^ T cells, but it was not observed in that by CD4^+^ T cells (Figure 5). Overall, there was a 13.7% reduction in CD8^+^ T cell proliferation (weighted average, ranging 0–49%).

## 4. Discussion

CL is characterized by one or multiple well-delimited cutaneous ulcers with raised borders [26]. Meglumine antimoniate is the more common therapy for ATL in Latin America and amphotericin is the second choice drug. However, while amphotericin B is associated with important side effects, a high rate of failure has been observed with antimony [27, 28]. Therefore, new forms of therapy for leishmaniasis are highly desirable. Considering that drugs are able to downmodulate the immune response in combination with a leishmanicidal agent, attenuate pathology, and enhance the cure rate in CL and ML patients, we evaluated in the present study the ability of DI4G, an inhibitor of PNP, to downmodulate the immune response in CL patients. The HsPNP inhibitor significantly decreased IFN-*γ* production *in vitro* and lymphocyte proliferation of leishmania antigen-stimulated CD8^+^ lymphocytes from CL patients.

The CL is characterized by an exaggerated production of proinflammatory cytokines produced by T cells and macrophages [5, 29]. There is a direct correlation between the frequency of CD4^+^ T cells expressing IFN-*γ* and the lesion size [8] and the frequency of CD8^+^ T cells expressing granzyme and the inflammatory reaction in CL ulcer [30]. Herein, we showed that DI4G decreased the production of IFN-*γ* in lymphocytes stimulated with leishmania antigen. The influence of this compound in IFN-*γ* was modest but significant. This is of particular interest for two reasons. First, because the exaggerated inflammatory response in CL is due to a decreased ability of regulatory cytokine to downmodulate the immune response. While the regulatory cytokine IL-10 decreased by 86% the IFN-*γ* production in lymphocyte cultures of tuberculin skin-test-positive healthy subjects stimulated with the *M. tuberculosis*-purified protein derivate (PPD), IL-10 decreased by only 48% the IFN-*γ* production by cells from CL patients stimulated with leishmania antigen [5]. Second, as IFN-*γ* is important for parasite killing, the downmodulation of this cytokine should be aimed to reduce pathology but preserving the host's ability to control parasite proliferation.

Despite the demonstration that DI4G decreased IFN-*γ* production, when we analyzed if this effect was in CD4^+^ T cells, CD8^+^ T cells, or NK cells, we did not observe any decrease of IFN-*γ* expression by these cells. As in CL, where one important source of IFN-*γ* production are double-negative lymphocytes [31], it is possible that the action of the DI4G was in CD4^−^ CD8^−^ double-negative T cells.

The pathogenesis of most of the infectious diseases is dependent on both the infectious agent and host factors. In protozoa infection, the role of the host immune response in producing tissue damage has been well documented in malaria [32], Chagas disease [33, 34], and ATL [13]. In such case, tissue damage has been associated to a hypersensitivity reaction or to an autoimmunity phenomenon [10, 35]. As a decrease in the immune response may favor bacteria, virus, protozoa, and helminthes proliferation, it is important that the modulation of the immune response attenuate pathology, but not impair the defense mechanism.

In ATL, evidences have been accumulated indicating that T cells and mainly CD8^+^ T cells participate in the pathology of the disease. High lymphocyte proliferation is also characteristic of CL, and we showed that DI4G decreased lymphocyte proliferation predominantly in CD8^+^ T cells rather than in CD4^+^ T cells. The role of inflammatory and cytotoxic CD8^+^ T cells in the pathology of CL has been well documented [7, 30, 36, 37]. More recently, evidence has been brought that CD8^+^ T cells play a key role not only in the inflammatory response and ulcer development but also in the appearance of metastatic lesions in CL [38]. Therefore, it is possible that an exaggerated activation of CD8^+^ T cell is more dangerous for the disease than the inflammatory reaction induced by monocytes and CD4^+^ T cells.

## 5. Conclusions

This study showed that DI4G, a transition-state analog inhibitor of HsPNP, exhibited an immunomodulatory action in IFN-*γ* production by cells from CL patients and decreased CD8^+^ T cell proliferation, indicating that this agent may be able to reduce pathology associated with *L. braziliensis* infection and can be tested in combination with antimony as a new therapeutic strategy for CL.

## References

[1] WHO (2014). Leishmaniasis: worldwide epidemiological and drug access update. http://www.who.int/leishmaniasis/burden/Country_profiles/en/.

[2] Miranda J. C., Reis E., Schriefer A. (2002). Frequency of infection of *Lutzomyia phlebotomines* with *Leishmania braziliensis* in a Brazilian endemic area as assessed by pinpoint capture and polymerase chain reaction. *Memórias do Instituto Oswaldo Cruz*.

[3] Bittencourt A. L., Barral A. (1991). Evaluation of the histopathological classifications of American cutaneous and mucocutaneous leishmaniasis. *Memórias do Instituto Oswaldo Cruz*.

[4] Soong L., Chang C. H., Sun J. (1997). Role of CD4+ T cells in pathogenesis associated with *Leishmania amazonensis* infection. *Journal of Immunology*.

[5] Bacellar O., Lessa H., Schriefer A. (2002). Up-regulation of Th1-type responses in mucosal leishmaniasis patients. *Infection and Immunity*.

[6] Xin L., Li Y., Soong L. (2007). Role of interleukin-1beta in activating the CD11c(high) CD45RB^−^ dendritic cell subset and priming *Leishmania amazonensis*-specific CD4^+^ T cells in vitro and in vivo. *Infection and Immunity*.

[7] Santos Cda S., Boaventura V., Ribeiro Cardoso C. (2013). CD8^+^ granzyme B^+^-mediated tissue injury vs. CD4^+^IFNgamma^+^-mediated parasite killing in human cutaneous leishmaniasis. *The Journal of Investigative Dermatology*.

[8] Antonelli L. R., Dutra W. O., Almeida R. P., Bacellar O., Carvalho E. M., Gollob K. J. (2005). Activated inflammatory T cells correlate with lesion size in human cutaneous leishmaniasis. *Immunology Letters*.

[9] Faria D. R., Gollob K. J., Barbosa J. (2005). Decreased in situ expression of interleukin-10 receptor is correlated with the exacerbated inflammatory and cytotoxic responses observed in mucosal leishmaniasis. *Infection and Immunity*.

[10] Carvalho L. P., Passos S., Schriefer A., Carvalho E. M. (2012). Protective and pathologic immune responses in human tegumentary leishmaniasis. *Frontiers in Immunology*.

[11] Scott P., Novais F. O. (2016). Cutaneous leishmaniasis: immune responses in protection and pathogenesis. *Nature Reviews Immunology*.

[12] Brodskyn C. I., Barral A., Boaventura V., Carvalho E., Barral-Netto M. (1997). Parasite-driven in vitro human lymphocyte cytotoxicity against autologous infected macrophages from mucosal leishmaniasis. *Journal of Immunology*.

[13] Cardoso T. M., Machado A., Costa D. L. (2015). Protective and pathological functions of CD8^+^ T cells in *Leishmania braziliensis* infection. *Infection and Immunity*.

[14] O'Neal S. E., Guimaraes L. H., Machado P. R. (2007). Influence of helminth infections on the clinical course of and immune response to *Leishmania braziliensis* cutaneous leishmaniasis. *The Journal of Infectious Diseases*.

[15] Llanos-Cuentas A., Tulliano G., Araujo-Castillo R. (2008). Clinical and parasite species risk factors for pentavalent antimonial treatment failure in cutaneous leishmaniasis in Peru. *Clinical Infectious Diseases*.

[16] Machado P. R., Lessa H., Lessa M. (2007). Oral pentoxifylline combined with pentavalent antimony: a randomized trial for mucosal leishmaniasis. *Clinical Infectious Diseases*.

[17] Santos J. B., de Jesus A. R., Machado P. R. (2004). Antimony plus recombinant human granulocyte-macrophage colony-stimulating factor applied topically in low doses enhances healing of cutaneous leishmaniasis ulcers: a randomized, double-blind, placebo-controlled study. *The Journal of Infectious Diseases*.

[18] Almeida R. P., Brito J., Machado P. L. (2005). Successful treatment of refractory cutaneous leishmaniasis with GM-CSF and antimonials. *The American Journal of Tropical Medicine and Hygiene*.

[19] Sadeghian G., Nilforoushzadeh M. A. (2006). Effect of combination therapy with systemic glucantime and pentoxifylline in the treatment of cutaneous leishmaniasis. *International Journal of Dermatology*.

[20] Lessa H. A., Machado P., Lima F. (2001). Successful treatment of refractory mucosal leishmaniasis with pentoxifylline plus antimony. *The American Journal of Tropical Medicine and Hygiene*.

[21] Bzowska A., Kulikowska E., Shugar D. (2000). Purine nucleoside phosphorylases: properties, functions, and clinical aspects. *Pharmacology & Therapeutics*.

[22] de Moraes M. C., Ducati R. G., Donato A. J. (2012). Capillary bioreactors based on human purine nucleoside phosphorylase: a new approach for ligands identification and characterization. *Journal of Chromatography. A*.

[23] Weirather J. L., Jeronimo S. M., Gautam S. (2011). Serial quantitative PCR assay for detection, species discrimination, and quantification of *Leishmania* spp. in human samples. *Journal of Clinical Microbiology*.

[24] Semeraro T., Lossani A., Botta M. (2006). Simplified analogues of immucillin-G retain potent human purine nucleoside phosphorylase inhibitory activity. *Journal of Medicinal Chemistry*.

[25] Soares A., Govender L., Hughes J. (2010). Novel application of Ki67 to quantify antigen-specific in vitro lymphoproliferation. *Journal of Immunological Methods*.

[26] Llanos Cuentas E. A., Cuba C. C., Barreto A. C., Marsden P. D. (1984). Clinical characteristics of human *Leishmania braziliensis* braziliensis infections. *Transactions of the Royal Society of Tropical Medicine and Hygiene*.

[27] Newlove T., Guimaraes L. H., Morgan D. J. (2011). Antihelminthic therapy and antimony in cutaneous leishmaniasis: a randomized, double-blind, placebo-controlled trial in patients co-infected with helminths and Leishmania braziliensis. *The American Journal of Tropical Medicine and Hygiene*.

[28] Brito G., Dourado M., Polari L. (2014). Clinical and immunological outcome in cutaneous leishmaniasis patients treated with pentoxifylline. *The American Journal of Tropical Medicine and Hygiene*.

[29] Giudice A., Vendrame C., Bezerra C. (2012). Macrophages participate in host protection and the disease pathology associated with *Leishmania braziliensis* infection. *BMC Infectious Diseases*.

[30] Faria D. R., Souza P. E., Duraes F. V. (2009). Recruitment of CD8^+^ T cells expressing granzyme A is associated with lesion progression in human cutaneous leishmaniasis. *Parasite Immunology*.

[31] Bottrel R. L., Dutra W. O., Martins F. A. (2001). Flow cytometric determination of cellular sources and frequencies of key cytokine-producing lymphocytes directed against recombinant LACK and soluble leishmania antigen in human cutaneous leishmaniasis. *Infection and Immunity*.

[32] Hunt N. H., Golenser J., Chan-Ling T. (2006). Immunopathogenesis of cerebral malaria. *International Journal for Parasitology*.

[33] Higuchi Mde L., Benvenuti L. A., Martins Reis M., Metzger M. (2003). Pathophysiology of the heart in Chagas’ disease: current status and new developments. *Cardiovascular Research*.

[34] Marin-Neto J. A., Cunha-Neto E., Maciel B. C., Simoes M. V. (2007). Pathogenesis of chronic Chagas heart disease. *Circulation*.

[35] Cunha-Neto E., Teixeira P. C., Nogueira L. G., Kalil J. (2011). Autoimmunity. *Advances in Parasitology*.

[36] Dantas M. L., Oliveira J. C., Carvalho L. (2013). CD8+ T cells in situ in different clinical forms of human cutaneous leishmaniasis. *Revista da Sociedade Brasileira de Medicina Tropical*.

[37] Novais F. O., Carvalho A. M., Clark M. L. (2017). CD8^+^ T cell cytotoxicity mediates pathology in the skin by inflammasome activation and IL-1beta production. *PLoS Pathogens*.

[38] Novais F. O., Carvalho L. P., Graff J. W. (2013). Cytotoxic T cells mediate pathology and metastasis in cutaneous leishmaniasis. *PLoS Pathogens*.

